# Pathogenicity and functional analysis of *CFAP410* mutations causing cone-rod dystrophy with macular staphyloma

**DOI:** 10.3389/fmed.2023.1216427

**Published:** 2023-10-12

**Authors:** Shaoqing Yang, Ya Li, Lin Yang, Qingge Guo, Ya You, Bo Lei

**Affiliations:** ^1^Henan University People’s Hospital, Henan Provincial People’s Hospital, Zhengzhou, China; ^2^Henan Branch of National Clinical Research Center for Ocular Diseases, Henan Eye Institute/Henan Eye Hospital, People’s Hospital of Zhengzhou University, Henan Provincial People’s Hospital, Zhengzhou, China

**Keywords:** *CFAP410*, CORD, compound heterozygous variants, cell cycle, ubiquitination

## Abstract

**Background:**

Cone-rod dystrophy (CORD) caused by pathogenic variants in *CFAP410* is a very rare disease. The mechanisms by which the variants caused the disease remained largely unknown. *CFAP410* pathogenic variants were identified in a cone-rod dystrophy with macular staphyloma patient. We explored the pathogenicity and performed functional analysis of two compound heterozygous mutations.

**Methods:**

A 6-year-old boy complained decreased vision for 1 year, underwent ocular examinations together with systemic X-ray check. Blood sample was taken for targeted next generation sequencing (Tg-NGS). Pathogenicity of identified variants was determined by ACMG guideline. Mutated plasmids were constructed and transferred to HEK293T cells. Cell cycle, protein stability, and protein ubiquitination level was measured.

**Results:**

The best-corrected visual acuity of proband was 0.20 bilaterally. Fundus showed macular staphyloma and uneven granular pigment disorder in the periphery of the retina. SS-OCT showed thinning and atrophy of the outer retina, residual ellipsoid zone (EZ) in the fovea. Scotopic and photopic ERG responses severe reduced. Two heterozygous missense pathogenic variants, c.319 T > C (p.Tyr107His) and c.347 C > T (p.Pro116Leu) in exon 4 of the *CFAP410*, were found and were pathogenic by the ACMG guideline. *In vitro*, pathogenic variants affect cell cycle. Immunofluorescence and western blotting showed that the mutant proteins decreased expression levels protein stability. Meanwhile, co-IP data suggested that ubiquitination level was altered in cells transferred with the mutated plasmids.

**Conclusion:**

Compound heterozygous pathogenic variants c.319 T > C and c.347 C > T in *CFAP410* caused CORD with macular staphyloma. The pathogenic mechanisms may be associated with alternations of protein stability and degradation through the ubiquitin-proteasome pathway.

## 1. Introduction

The cone-rod dystrophies (CORDs) are subgroups of inherited retinal diseases (IRDs) and can be inherited through AR, AD, or XL (1). The prevalence is about 1/20,000–100,000 (1, 2). Victims of CORDs suffer from progressive vision loss, photophobia, and color vision abnormalities in childhood or early adulthood (3). CORDs are characterized by both cone and rod photoreceptor abnormality, which can be distinguished from cone dystrophy by involvement of rod dysfunction (2). CORDs are known to be either isolated or part of a systemic disease. So far at least 22 genes have been reported to be associated with isolated CORDs (4).

**Figure 1:**
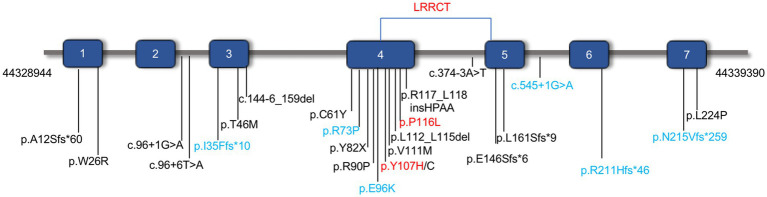
The distribution of identified pathogenic variants in *CFAP410* (NM_004928.2). Pathogenic variants associated with isolated CORD or RP were shown in black and those associated with syndromic retinal degeneration AXSMD or JATD were in blue. The pathogenic variants verified in this study were in red.

Initially, *CFAP410* was studied as a causative gene for Alzheimer’s disease (5) and was later found to be associated with retinal ciliopathy (6) or syndromic ciliopathy. Some pathogenic variants in *CFAP410* caused isolated retinal disorders with or without macular staphyloma, such as RP, CORD (6–20) (Figure 1). Pathogenic variant have also been reported in patients with syndromic retinal degeneration such as AXSMD (9, 21–23), JATD (24) (Figure 1). The *CFAP410* gene (Cilia and Flagella Associated Protein 410, previously known as *C21orf2*), encodes a cilia-associated protein, it has been found in the base of the connecting cilium in the mouse photoreceptors (16, 25). Previous studies have demonstrated that loss of *CFAP410* in mammalian cells impaired the cilia formation and/or maintenance (26). CFAP410 was found to belong to the same complex with NEK1 and SPATA7. These proteins interact with each other in lysates of bovine retina (19). The detailed role of this protein in the production of cilia and mechanisms, however, has not been clearly investigated. In addition, this protein plays a key role in the repairment of DNA damage (27), and in regulation of cell morphology and cytoskeletal organization (28).

In this study, the phenotype of early-onset CORD with macular staphyloma in one individual from a non-inbred Chinese family was documented. The proband had two pathogenic variants (c.319 T > C, Tyr107His; c.347C > T, p.Pro116Leu) in *CFAP410* gene. After getting the genetic test results, we did check the patient carefully based on the pathogenic variants. No other systemic abnormalities were noticed. Although heterozygous missense variants were reported in patient with axial spondylometaphyseal dysplasia (AXSMD), function of these *CFAP410* pathogenic variants was less documented. After confirming the pathogenicity of P116L and Y107H in this family, we tested the hypothesis that the variants affected protein instability and increased degradation of the functional protein.

## 2. Materials and methods

### 2.1. Subject and clinical evaluation

This study complied with the Helsinki declaration and was approved by the Ethics Committee of Henan Provincial Eye Hospital for the publication of clinical information, family history and blood extraction for genetic testing [HNEECKY-2019(15)] with the consent of all subjects. Detailed ophthalmic examination including best corrected visual acuity (BCVA), fundus photography, swept source optical coherence tomography (SS-OCT, VG200D SVision Imaging, Henan, China), and full-field electroretinography (ERG, ROLAND CONSULT, Brandenburg, Germany) with skin electrodes. To exclude the possibility that multi-organ was affected, skeletal survey and chest *x*-ray were then examined. Other members of the family line also underwent BCVA, SS-OCT, and other ocular examinations.

### 2.2. Targeted next-generation sequencing

Genomic DNA of subjects was extracted from peripheral blood. Targeted next-generation sequencing (Tg-NGS) of DNA using a custom-designed panel (PS400) which contains 376 known genes involved in inherited retinal diseases, including the exons and adjacent intron regions (50 bp) and known intron mutation (29). The captured DNA was sequenced using a high-throughput sequencer (Illumina) after elution, amplification, and purification. Sequencing data were aligned with the human genome reference (UCSC hg19) using TGex (LifeMap Science, Alameda, United States), Efficient Genosome Intepration System, EGIS (SierraVast Bio-Medical, Shanghai, China), and XY Gene Ranger 2.0 software (Seekgene, Shanghai, China) and genetic variants were identified. Quality parameters such as coverage of target regions and average sequencing depth were collected. The average sequencing depth of the target region for retinal genetic disease-associated gene detection is 200X. Sanger sequencing and family segregation analysis were used to verify suspected disease-associated gene variants in available family members.

### 2.3. *In silico* analysis

SIFT, Polyphen2, LRT, Mutation Taster, CADD, and others were used to predict the pathogenicity of the mutation and gnomAD, ExAC, 1,000 genomes was used to determine allele frequencies of the variants in East Asian populations (30). Clustal Omega and WebLogo were employed for sequence comparison between different species. AphalFold was used to be predicted tertiary structure of protein. Predicting and analyzing changes in the structure and function of mutant proteins were performed by using the PyMoL and HOPE online software. CFAP410 mutant protein stability prediction with the online tool I-Mutant v2.0 and MUpro (31).

### 2.4. Cell culture

The HEK293T cells used in this experiment were purchased from American Type Culture Collection (ATCC, Manassas, VA, United States). Cells were grown in medium containing DMEM of high glucose (SH30022.01, HyClone, UT, United States), 10% fetal bovine serum (35-081-CV, Corning, NY, United States), and 1% penicillin–streptomycin (32105, Mengbio, Chongqing, China). The conditions of the incubator are 37°C, 5% CO_2_.

### 2.5. Plasmid and transfection

Sangon Biotech (Shanghai, China) was commissioned to synthesize CDS of *CFAP410* (NM_004928.3) wild-type, *CFAP410* c. 319 T > C and *CFAP410* c.347C > T containing a C-terminal Flag and clone into the vector separately. The constructed vectors were confirmed by Sanger sequencing (Sangon Biotech). Well-grown cells with a density of 80% were transfected with EZ Cell Transfection Regent (Life-iLab, Shanghai, China) according to the manufacturer’s instructions.

### 2.6. Western blotting

Cells were lysed with RIPA (PC101, EpiZyme, Shanghai, China), supplemented with 1% protease inhibitor cocktail (GRF101, EpiZyme). Cell lysates were denatured and separated by 12.5% SDS-PAGE electrophoresis, and transferred to polyvinylidene fluoride membranes (IPFL00010, Millipore, MA, United States). The membranes were blocked with 5% skim milk and incubated with primary antibodies mouse anti-Flag (1:5,000; 2367S, Cell Signaling Technology, MA, United States) and rabbit anti-βactin (1:5,000; 4970S, Cell Signaling Technology) at 4°C overnight. After washing with PBS containing 0.1% Tween-20 (PBST), membranes were incubated with secondary antibodies at room temperature for 2 h and visualized with Chemiluminescent detection reagent (WBKLS0500, Millipore). ImageJ software (Version 1.52a, NIH) was used to analyze bands.

### 2.7. Protein stability assay

After 24 h cell culture, HEK293T cells transfected with pcDNA3.1, Flag-*CFAP410*, Flag-*CFAP410* c.319 T > C, and Flag-*CFAP410* c.347C > T were treated with 100 μM cycloheximide (CHX, HY-N0901, MedChemExpress, NJ, United States) or CHX mixed inhibitors of proteasome (MG132). Cells were scraped at 0, 6, 12, and 18 h after CHX/MG132 exposure using ice-cold PBS. The total protein was extracted for Western blot. Wild-type and mutant CFAP410 protein levels were detected using flag antibody. β-actin were the loading control (32).

### 2.8. Co-immunoprecipitation assay

HEK293T cells were transfected with 12 μg of Flag-*CFAP410* WT plasmid, Y107H and P116L plasmids in 10 cm plates that were 80% full of cells, and harvested for protein extraction after 24 h. Total protein lysate was extracted by immunoprecipitation buffer (BL509A, Biosharp, Guangzhou, China), and the concentration of the protein was quantified with BCA protein assay kit (P0011, Beyotime, Shanghai, China). 1,000 μg proteins were mixed with 10 μg anti-Flag magnetic beads (HY-K0207, MedChemExpress) and shaken at 4°C on rotor overnight. Beads with absorbed proteins were gathered by Magnetic Stand (HY-K0200, MedChemExpress) and washed three times by IP-buffer containing 1% protease inhibitor. The beads added to 1× loading buffer boiled for 10 min and the supernatant was collected. The analyzed by western blot.

### 2.9. Cell-cycle analysis

Cells were divided into the following four groups: pcDNA3.1, WT, Y107H, and P116L. The cells were seeded into six-well tissue culture plates (4 × 10^5^ cells/well). After transfection for 24 h, the cells were collected and washed with 1 × PBS. Added pre-cooled ethanol (concentration 75%) while vortexing on a turbo shaker and refrigerate for more than 12 h. Cells washed with PBS and then 0.5 mL PI/RNase Staining Buffer (550,825, BD, NJ, United States) was added and cells were incubated for 30 min at room temperature. The DNA content was detected using flow cytometry (Canto plus, BD). The percentage of cells in the G1 phase, the S phase, and the G2 phase was analyzed.

### 2.10. Immunofluorescence

Cell crawls were fixed with 4% paraformaldehyde for 30 min at room temperature, washed with PBS and permeabilized with 0.5% Triton X-100 (in PBS) for 20 min. Slides were incubated with 5% bovine serum albumin for 2 h at room temperature and then incubated with anti-mouse IgG-FITC antibodies (1:250; abs20004, Absin, Shanghai, China) overnight at 4°C. After being washed three times with PBS, the secondary anti-mouse Alexaflor 594 (1:250; abs20017, Absin) were applied for 2 h at room temperature in the dark. Nuclei were counterstained with DAPI (F6057, Sigma, MO, STL, United States). Luorescence microscopy images were obtained by Zeiss confocal microscope (NLO780, Zeiss, Oberkochen, Germany).

### 2.11. Statistical analysis

Statistical software GraphPad Prism was used to analyze the experimental data, and one-way ANOVA was used for multiple comparisons. *p* < 0.05 was considered statistically significant.

## 3. Results

### 3.1. Clinical features

**Figure 2:**
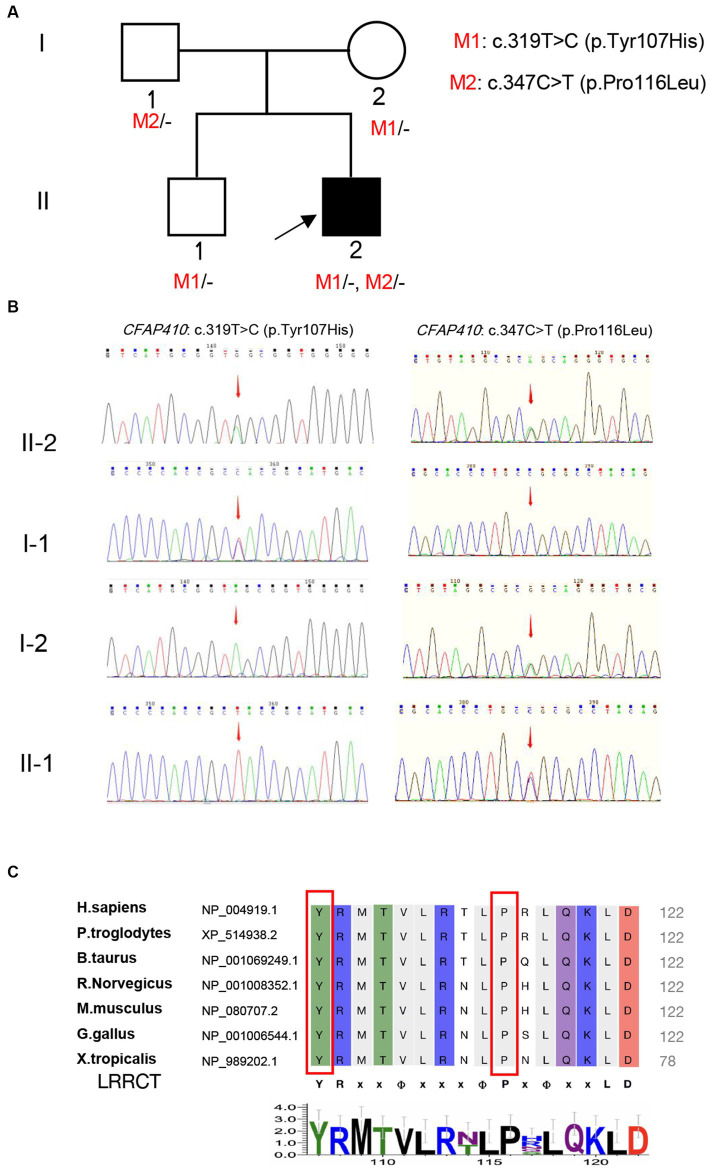
Validation and predictive analysis of the pathogenic variants. **(A)** Pedigree of family. M1: *CFAP410* c.319 T > C, M2: *CFAP410* c.347C > T; **(B)** Sanger sequencing results in the family members. Sequencing results showed that pathogenic variant *CFAP410* c.347C > T was not detected in the proband’s father **(I-1)**, but variant c.319 T > C was detected, indicating that the variant in the patient came from father; The pathogenic variant *CFAP410* c.319 T > C was not detected in the proband’s mother **(I-2)**, but c.347C > T was detected, indicating the variant in the patient was derived from the mother. The patient’s older brother only carried the variant *CFAP410* c.347C > T. **(C)** Multiple alignments of Tyr107 and Pro116 in CFAP410 protein among different species. LRRCT was shown below the alignment (YRxxΦxxxΦPxΦxxLD). “Φ” represented a hydrophobic residue, “*x*” represented unfixed amino acid.

**Figure 3:**
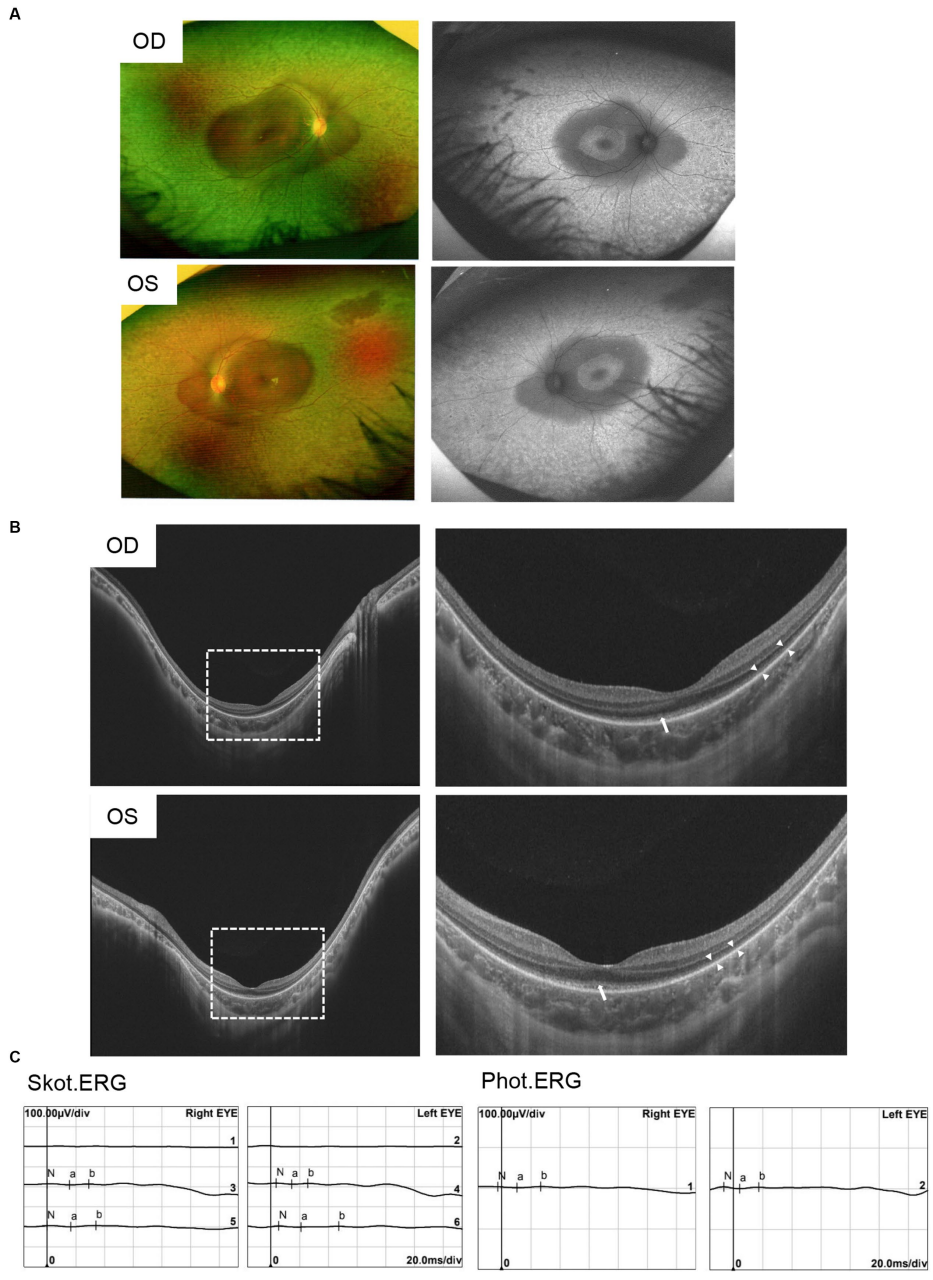
Clinical characteristics of patients with pathogenic variants in *CFAP410.* **(A)** Ophthalmic symptoms of the proband. Fundus photograph showed uneven granular pigment disorder in the periphery of the retina. Fundus autofluorescence showed markedly hyperautofluorescence in the posterior pole and in the peripheral retina area in both eyes. **(B)** SS-OCT showed macular staphyloma, atrophy and thinning of the outer retina, and residual ellipsoid zone in the fovea of the macula in both eyes. “Long arrows” indicated the residual EZ and “short arrows” indicated the atrophied outer retina. **(C)** ERG showed severe reduced rod- and cone-mediated responses.

**Figure 4:**
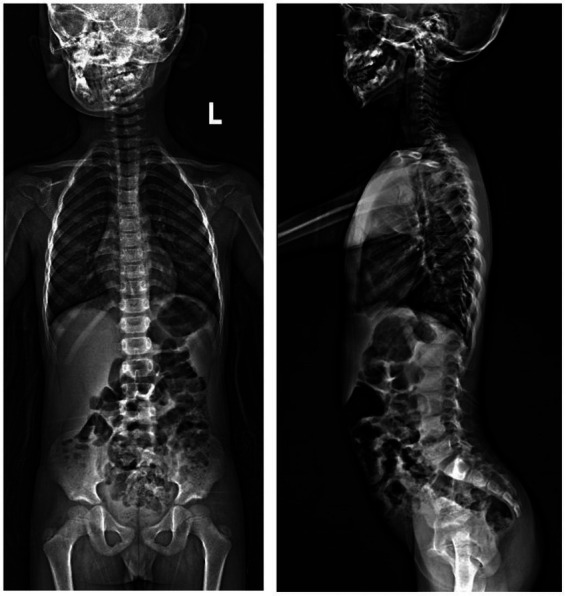
X-ray photographs showed no significant abnormalities of skeleton.

A Chinese family was identified and the pedigree was shown in Figure 2A. The proband, a 6-year-old boy, was noted to have had slowly progressive vision loss and narrow visual field in both eyes since 1 year ago. Night blindness was denied by boy’s parents. At presentation, the best-corrected visual acuity was 0.20 (+0.50/−1.25 × 20) of right eye and 0.20 (+1.00/−2.00 × 170) of left eye. The proband’s right and left eye axial length were 22.23 mm and 22.43 mm, respectively. There were no nystagmus or strabismus. Fundus showed macular staphyloma and uneven granular pigment disorder in the periphery of the retina (Figure 3A). Fundus autofluorescence in both eyes showed an enhanced ring-shape fluorescence in the posterior pole under a low fluorescence background and hyperfluorescence in the peripheral retina. SS-OCT displayed thinning and atrophy of the outer retina, missing of ellipsoid zone (EZ) and interdigitation zone (IZ), with only a small amount of EZ remained in the fovea. In addition, macular staphyloma was noted in both eyes (Figure 3B). Full-field ERG revealed severe reduction in scotopic and photopic ERG responses (Figure 3C). Other examinations including pupils and anterior segments were normal. The proband presented no hearing abnormalities. The chest X-ray showed no significant skeletal abnormalities (Figure 4). The boy’s height and weight were in normal range. Cardiac ultrasound showed no significant abnormalities. No other developmental problems were noticed.

None of remaining family members showed abnormalities related to body development, stature, or ocular history (Supplementary Figure 1).

### 3.2. Genetic analysis revealed two missense variants in *CFAP410*

A total of 12 variants in 11 genes were detected in the blood samples of the proband by Tg-NGS. Except for *CFAP410*, which contained two heterozygous missense pathogenic variants, only one variant was detected in the remaining 10 genes, and the pathogenicity was unknown. Most of the unrelated variants were excluded (Supplementary Table 1). Two heterozygous missense pathogenic variants, c.319 T > C (p.Tyr107His) and c.347C > T (p.Pro116Leu), in exon 4 of the *CFAP410* located at chr.21–45,752,942 and chr.21–45,752,970, were considered to be phenotypically related. Sanger sequencing revealed that the variants segregated in participating individuals and followed an autosomal recessive heritability pattern (Figure 2B).

Y107H was the relatively common pathogenic variant reported in *CFAP410*, and its pathogenicity has also been verified *in vitro*. Functional experiments showed that this variant could lead to reduction of protein expression and localization of proteins in cells (16). Recently, homozygous variant Y107H was found in two patients with RP and ALS (33). Y107C, tyrosine changed into cysteine at position 107, has been also reported to cause isolated RP or CORD in homozygous patients (12, 16). According to ACMG guidelines, *CFAP410* c.319 T > C (p.Tyr107His) was “pathogenic” (PS1 + PS3 + PM1 + PP1 + PP3).

Another missense pathogenic variant, P116L, was also located in the same LRRCT (leucine-rich repeat C-terminal) structural domain as Y107H and was highly conserved in interspecies amino acid sequence comparisons (Figure 2C). Mutation of a 100% conserved residue is usually damaging for the protein. The P116L was predicted to be harmful by several *in silico* tools (Supplementary Table 2). According to ACMG guidelines, this variant was “likely pathogenic” (PS3 + PM1 + PP1 + PP3).

### 3.3. Prediction of protein structure

**Figure 5:**
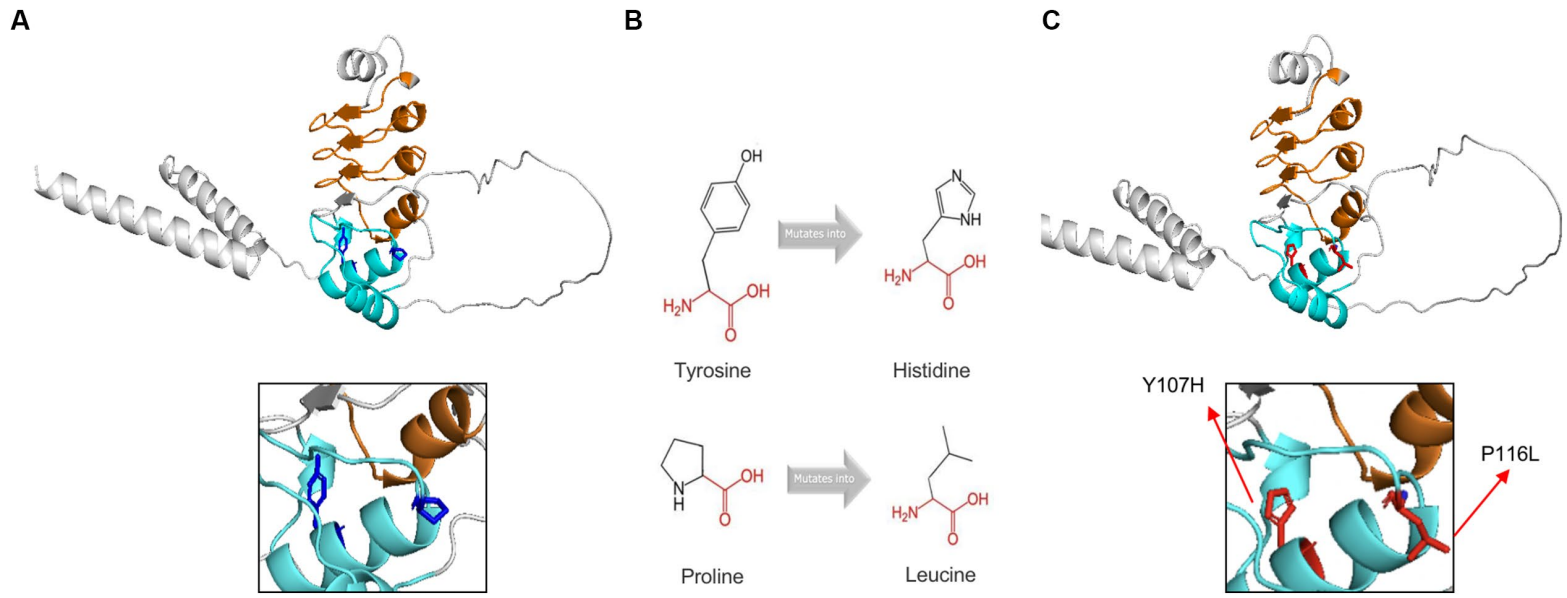
Tertiary structure prediction of mutant proteins. The main structure of the CFAP410 protein was colored in gray. Leucine-rich repeats was in orange and LRRCT was in blue. **(A)** The side chains of the wild-type were colored in blue. **(B)** The schematic structures of the original and the mutant amino acids. The backbone was colored in red; The side chain was colored in black. **(C)** The side chains of the wild-type were colored in red.

The exact 3D-structure of *CFAP410* was unknown. We predicted protein model by AlphaFold (34, 35) and the predicted structural changes of the mutant proteins were shown in Figure 5. To predict the effects of pathogenic variants on protein structure and function, we used the online software HOPE. Compared with the wild-type residue, the mutant residue at position 107 was smaller and more hydrophilic (Figure 5B). This change might cause loss of hydrophobic interactions with other molecules on the surface of the protein. The variant residue at position 116 was bigger, which might lead to bumps (Figure 5B). LRRCT is critical for the folding of proteins (36), thus alternations in sizes and properties of amino acid may lead to changes in stability. Indeed, results obtained from the online tool I-Mutant v2.0 suggested that the stability of the mutant *CFAP410* proteins decreased. However, prediction software MUpro showed increased stability.

### 3.4. Two pathogenic variants prolonged G1 phase

Ciliogenesis is closely related to the cell cycle. It was shown that *NEK1*, a causative gene of ciliopathy, was required in regulating cell cycle (37). CFAP410 and NEK1 belonged to the ciliary functional modules and played a consistent role in ciliogenesis.

**Figure 6:**
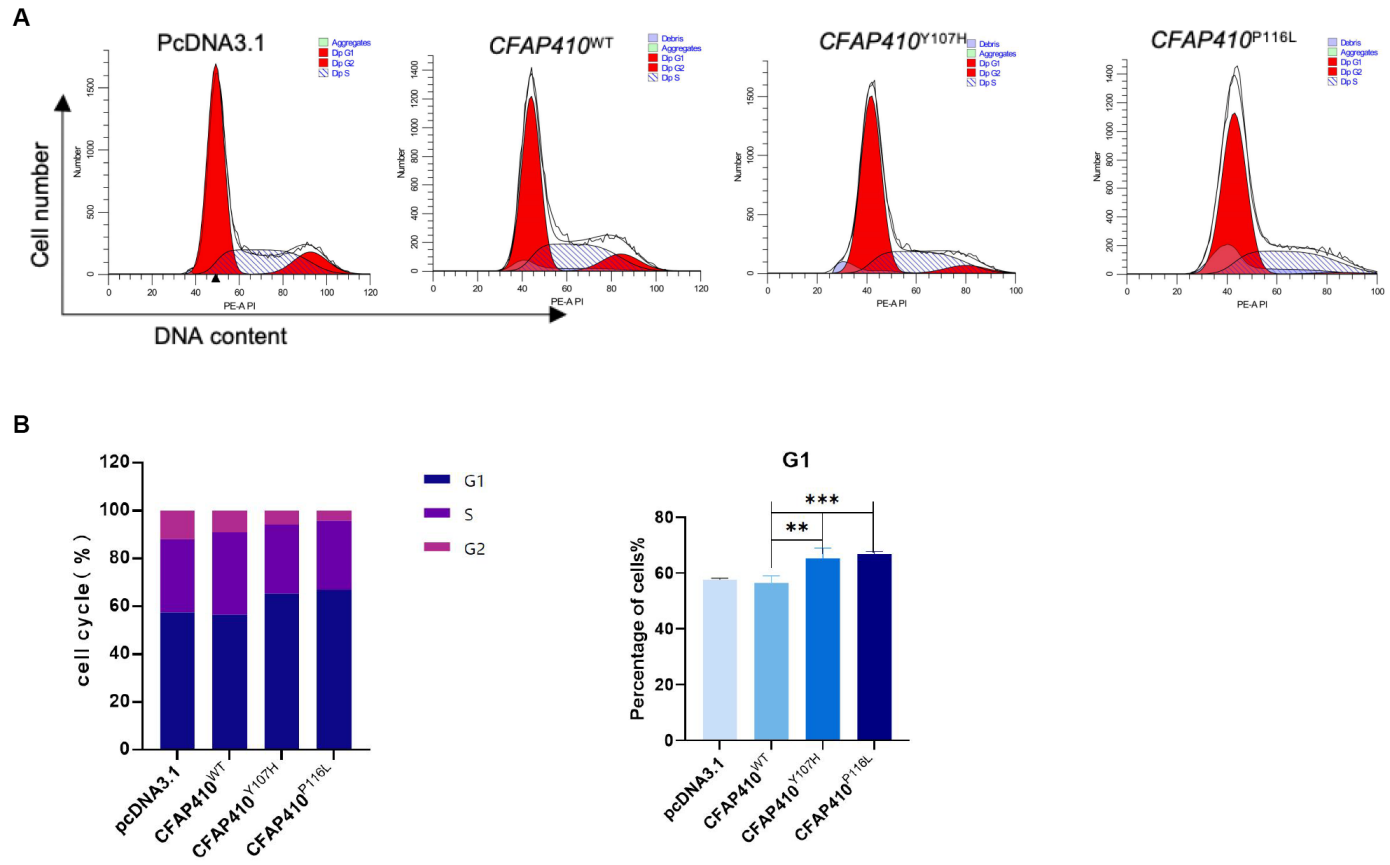
Effect of variants on the cell cycle in mutant transferred HEK293T cells. **(A)** After 24 h transfection with plasmids of with wild-type, Y107H, and P116L, cycle distribution was detected. **(B)** The proportion of cells in different cell cycles in each group was used to make a statistical chart. Error bars indicate means ± SD, *n* = 3, ^**^p < 0.01, ^***^p < 0.001.

Therefore, we verified the effect of mutant proteins on cell cycle. We constructed wild-type (*CFAP410*-WT), mutant (*CFAP410*-P116L and *CFAP410*-Y107H) plasmids using pcDNA3.1 as vectors and transfected them into HEK293T cells. 24 h after transfection with plasmids into cultured cells. G1 phase were blocked in the cell cycle in the two pathogenic variant groups when compared with the empty vector (pcDNA3.1) and wild-type group (Figure 6).

### 3.5. Two pathogenic variants damaged the stability of CFAP410 protein

Pathogenic variant located in the LRRCT may affect the stability of the protein and I-Mutant v2.0 online tool predicted reduction in the stability of the mutant proteins.

**Figure 7:**
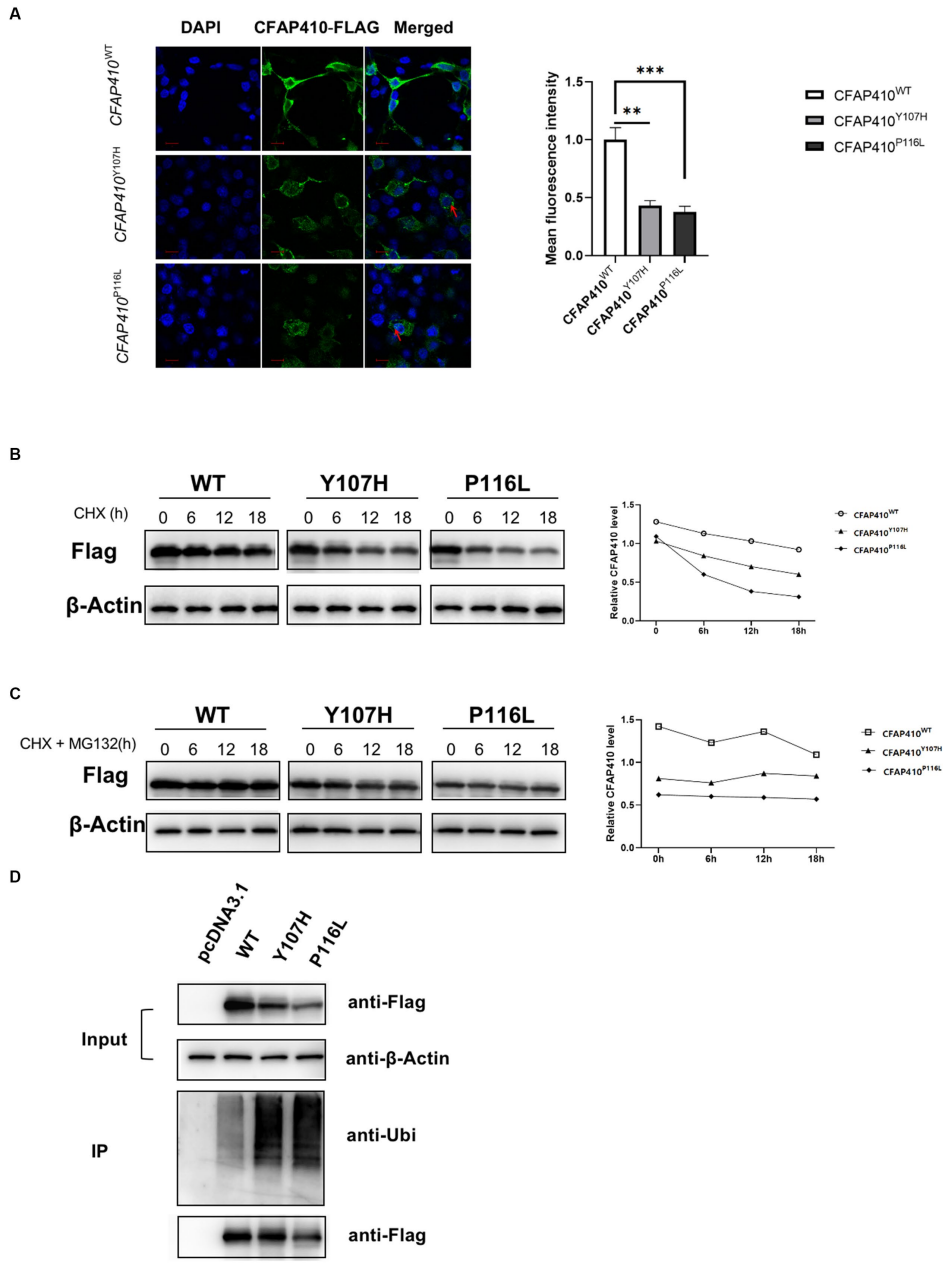
Pathogenic variant damaged the stability of CFAP410 protein may through the ubiquitination-proteasome pathway. **(A)** The expression of wild-type, Y107H and P116L CFAP410 proteins in HEK293T cells. CFAP410 were labeled with anti-Flag (green). DAPI (blue) was used to stain nuclei. The red arrows marked the clusters of proteins (*n* = 3, ^**^p < 0.01, ^***^p < 0.001). **(B)** HEK293T cells were transfected with wild-type, Y107H and P116L mutant plasmids for 18 h, and treated with 100 μM CHX, the expression of CFAP410 protein was detected. **(C)** CFAP410 protein stability was detected after cells were treated with 50 μM MG132. **(D)** 24 h after transfection, wild-type, Y107H, and P116L proteins were enriched by immunoprecipitation, and their ubiquitination levels were detected.

To determine changes in mutant protein content, immunofluorescence experiments were performed. Fluorescence intensity of the mutants Y107H and P116L transferred cells was significantly weaker than that of the wild type (Figure 7A). We found that the mutant proteins were unevenly distributed in the cytoplasm and some of the proteins were presented in clusters.

We then performed CHX assay to verify the changes in protein stability. At 18 h after plasmid transfection, HEK293T cells in each group were treated with 100 μM CHX for 0, 6, 12, and 18 h. Western blot showed that the half-life of CFAP410 protein carrying the Y107H variant and P116 L variant was significantly shorter compared with the wild-type protein, indicating that both pathogenic variants impaired the stability of CFAP410 protein (Figure 7B). The ubiquitin-proteasome system is essential for maintaining protein homeostasis at the level of protein degradation. We added MG132, a protease inhibitor, to CHX-treated cells. The results showed a significant increase in protein stability of the *CFAP410* mutants transferred cells (Figure 7C). Subsequently, we examined the ubiquitination levels of CFAP410 proteins in HEK293T cells. We found that the ubiquitination levels of P116L and Y107H proteins were significantly increased compared to the wild-type CFAP410 proteins (Figure 7D). The data suggested that the reduced stability of the two mutant proteins might be associated with the ubiquitin-proteasome pathway.

## 4. Discussion

Pathogenic variant located in the LRRCT domain may cause retinal ciliopathy by reducing functional protein through misfolding (16). The pathogenicity of this important domain in retinal ciliopathy has been emphasized in a recent report (38). In this study, we identified compound heterozygous missense pathogenic variants in *CFAP410* (NM_004928.2: c.319 T > C, p.Tyr107His and c.347C > T, p.Pro116Leu) in a Chinese boy diagnosed with CORDs with macular staphyloma. The two pathogenic variants happened to be located in the LRRCT domain.

Patients suffering from retinal ciliopathy typically present with early onset of visual symptoms, which was consistent with the symptoms of the patient in this study. Proband presented with severe loss of vision at 6 years old. The same heterozygous missense variants in *CFAP410* were previously reported with AXSMD in a Korean family (23). However, the clinical features of the Korean patients were different from this boy. Although both patients had ocular manifestations, retinal dystrophy appeared earlier and was more severe in our study. Nevertheless, skeletal manifestations were more pronounced in the Korean patients, including short stature, thoracic stenosis, upper limb rhizomelic shortening, lacy ilia, and metaphyseal dysplasia of proximal femur was evident. While, skeletal abnormalities and short stature were not detected in this Chinese boy. Given the absence of characteristic skeletal manifestations, diagnosis of AXSMD was excluded. The different phenotypes presented in this study and previous reports suggested the intrinsic mechanisms may be complex.

The pathogenicity of Y107H has been demonstrated. Our data further verified the effects of the two pathogenic variants on the encoded protein. In addition to the findings that two variants reduced the function of the proteins, we further showed variants increased protein degradation *via* the ubiquitin-proteasome pathway and affected the cell cycle.

It has been established that *CFAP410* is located in the connecting cilia of mouse and monkey photoreceptors (16, 19). A small-scale siRNA screen showed that knockdown of *CFAP410* impairs cilia formation through Hedgehog (Hh) signaling in mammalian cells *in vitro* (26). The outer segments (OS) of rod and cone photoreceptors are specialized sensory cilia that have an important role in converting light signals to neural electrical signals (39–41). Consequently, abnormal cilia function or structure may cause photoreceptor degeneration, which in turn leads to retinal dystrophy (42). It has been shown that pathogenic variants in *CFAP410* resulting in reduced protein expression may have a significant effect on cilia formation or maintenance in photoreceptor cells (16). Furthermore, primary cilia are widely distributed in all vertebrate cell, such as the retina, central nervous system, liver, kidney, and skeletal system (43). Abnormal ciliary function may also cause developmental disorders in these systems. Although the presence of *CFAP410* has not been confirmed in the cilia of other organs, it may affect skeletal development based on the clinical presentation of some patients with syndromic ciliopathies.

The cell cycle is a very conservative process in evolution and includes G0/G1, S, G2, and M phases. Cells remain quiescent during G0 phase, G1 phase for organelle generation to begin transcription and translation, S phase for DNA replication, G2 phase for DNA replication to complete in preparation for cell division and cells split in M phase (44). The processes ciliary growth and absorption are closely related to the cell cycle, present in G0/G1 phase, reabsorbed before the onset of mitosis, and reappear after the cell splits (45). In addition, cell cycle may affect some proteins that regulate ciliogenesis. For example, CP110 is a protein that negatively regulates cilia assembly and its protein amount is significantly decreased in G2/M phase and G0/G1 phase (46). By analyzing the number of transferred HEK293T cells in different cycle phases, we found that the G1 phase was blocked in the mutant P116L and Y107H groups compared to the wild type group. We speculated that subtle changes in cell cycle progression might have direct or indirect effects on cilia development.

A relatively stable protein amounts is important for organisms to maintain cellular morphology and function. Pathogenic variants causing RD were observed to decrease the amount of protein in cells *in vitro* (16). Another pathogenic variants causing ALS may lead to the accumulation of *CFAP410* protein in motor neurons and induce the growth of neurites (47). In our experiments, immunofluorescence assays showed that the amount of fluorescence of P116L and Y107H transferred cells was significantly reduced compared to the wild type. The protein content of the mutant phenotype became unstable with the increasing time. Co-IP analysis showed increased degradation of the mutant protein via the ubiquitin-proteasome pathway. Thus, the normal function of *CFAP410* protein in ciliated cells was affected, the misfolded proteins may be preferentially degraded by the proteasome, and the ciliated cells were dysfunctional, affecting growth and development of different systems.

## 5. Conclusion

In summary, our results extended the phenotype associated with *CFAP410* variants. *CFAP410* pathogenic variants Y107H and P116L were associated with isolated cone-rod dystrophy with macular staphyloma. We speculated that decreased stability and increased degradation of the mutant proteins, as well as the impact on the cell cycle, might be responsible for the photoreceptor degeneration. Given the experimental limitations, the exact mechanisms deserve further investigations.

## Data availability statement

The datasets presented in this study can be found in LOVD repositories. The names of the repository/repositories and accession number(s) can be found below: https://databases.lovd.nl/shared/screenings/0000436512.

## Ethics statement

The study was conducted in accordance with the Declaration of Helsinki, and approved by the Institutional Review Board (or Ethics Committee) of Ethics Committee of Henan Eye Hospital [IRB approval number: HNEECKY-2019 (15); HNEECKY-2019-12-03]. The studies were conducted in accordance with the local legislation and institutional requirements. Written informed consent for participation in this study was provided by the participants’ legal guardians/next of kin. Written informed consent was obtained from the minor(s)' legal guardian/next of kin for the publication of any potentially identifiable images or data included in this article.

## Author contributions

BL: conceptualization. SY, YL, QG, YY, and LY: methodology. SY, BL, LY, and QG: writing—review and editing. All authors contributed to the article and approved the submitted version.

## References

[1] Hamel CP. Cone rod dystrophies. Orphanet J Rare Dis. (2007) 2:7. doi: 10.1186/1750-1172-2-7 17270046 PMC1808442

[2] Roosing S Thiadens AAHJ Hoyng CB Klaver CCW den Hollander AI Cremers FPM. Causes and consequences of inherited cone disorders. Prog Retin Eye Res. (2014) 42:1–26. doi: 10.1016/j.preteyeres.2014.05.001 24857951

[3] Tsang SH Sharma T. Progressive cone dystrophy and cone-rod dystrophy (XL, AD, and AR) In: Tsang SH Sharma T, editors. Atlas of Inherited Retinal Diseases Advances in Experimental Medicine and Biology. Cham: Springer International Publishing (2018). 53–60. 10.1007/978-3-319-95046-4_12 30578485

[4] Gill JS Georgiou M Kalitzeos A Moore AT Michaelides M. Progressive cone and cone-rod dystrophies: clinical features, molecular genetics and prospects for therapy. Br J Ophthalmol. (2019) 103:711–20. doi: 10.1136/bjophthalmol-2018-313278, PMID: 30679166 PMC6709772

[5] Shim KS Bergelson JM Furuse M Ovod V Krude T Lubec G. Reduction of chromatin assembly factor 1 p60 and C21orf2 protein, encoded on chromosome 21, in down syndrome brain In: Lubec G, editor. Advances in Down Syndrome Research Journal of Neural Transmission Supplement. Vienna: Springer Vienna (2003). 117–28. 10.1007/978-3-7091-6721-2_10 15068244

[6] Abu-Safieh L Alrashed M Anazi S Alkuraya H Khan AO al-Owain M. Autozygome-guided exome sequencing in retinal dystrophy patients reveals pathogenetic mutations and novel candidate disease genes. Genome Res. (2013) 23:236–47. doi: 10.1101/gr.144105.112, PMID: 23105016 PMC3561865

[7] Birtel J Gliem M Mangold E Müller PL Holz FG Neuhaus C. Next-generation sequencing identifies unexpected genotype-phenotype correlations in patients with retinitis pigmentosa. PLoS One. (2018) 13:e0207958. doi: 10.1371/journal.pone.0207958, PMID: 30543658 PMC6292620

[8] Carss KJ Arno G Erwood M Stephens J Sanchis-Juan A Hull S. Comprehensive rare variant analysis via whole-genome sequencing to determine the molecular pathology of inherited retinal disease. Am J Hum Genet. (2017) 100:75–90. doi: 10.1016/j.ajhg.2016.12.003, PMID: 28041643 PMC5223092

[9] de Castro-Miró M Tonda R Escudero-Ferruz P Andrés R Mayor-Lorenzo A Castro J. Novel candidate genes and a wide Spectrum of structural and point mutations responsible for inherited retinal dystrophies revealed by exome sequencing. PLoS One. (2016) 11:e0168966. doi: 10.1371/journal.pone.0168966, PMID: 28005958 PMC5179108

[10] Fadaie Z Whelan L Ben-Yosef T Dockery A Corradi Z Gilissen C. Whole genome sequencing and in vitro splice assays reveal genetic causes for inherited retinal diseases. NPJ Genom Med. (2021) 6:97. doi: 10.1038/s41525-021-00261-1, PMID: 34795310 PMC8602293

[11] Huang L Xiao X Li S Jia X Wang P Sun W. Molecular genetics of cone-rod dystrophy in Chinese patients: new data from 61 probands and mutation overview of 163 probands. Exp Eye Res. (2016) 146:252–8. doi: 10.1016/j.exer.2016.03.015, PMID: 26992781

[12] Jauregui R Chan L Oh JK Cho A Sparrow JR Tsang SH. Disease asymmetry and hyperautofluorescent ring shape in retinitis pigmentosa patients. Sci Rep. (2020) 10:3364. doi: 10.1038/s41598-020-60137-9, PMID: 32098976 PMC7042348

[13] Kameya S Fujinami K Ueno S Hayashi T Kuniyoshi K Ideta R. Phenotypical characteristics of POC1B -associated retinopathy in Japanese cohort: cone dystrophy with Normal funduscopic appearance. Invest Ophthalmol Vis Sci. (2019) 60:3432–46. doi: 10.1167/iovs.19-26650, PMID: 31390656

[14] Lionel AC Costain G Monfared N Walker S Reuter MS Hosseini SM. Improved diagnostic yield compared with targeted gene sequencing panels suggests a role for whole-genome sequencing as a first-tier genetic test. Genet Med. (2018) 20:435–43. doi: 10.1038/gim.2017.119, PMID: 28771251 PMC5895460

[15] Rodríguez-Muñoz A Aller E Jaijo T González-García E Cabrera-Peset A Gallego-Pinazo R. Expanding the clinical and molecular heterogeneity of nonsyndromic inherited retinal dystrophies. J Mol Diagn. (2020) 22:532–43. doi: 10.1016/j.jmoldx.2020.01.003, PMID: 32036094

[16] Suga A Mizota A Kato M Kuniyoshi K Yoshitake K Sultan W. Identification of novel mutations in the LRR-cap domain of C21orf2 in Japanese patients with retinitis pigmentosa and cone–rod dystrophy. Invest Ophthalmol Vis Sci. (2016) 57:4255–63. doi: 10.1167/iovs.16-19450, PMID: 27548899

[17] Wang L Zhang J Chen N Wang L Zhang F Ma Z. Application of whole exome and targeted panel sequencing in the clinical molecular diagnosis of 319 Chinese families with inherited retinal dystrophy and comparison study. Gene. (2018) 9:360. doi: 10.3390/genes9070360, PMID: 30029497 PMC6071067

[18] Weisschuh N Obermaier CD Battke F Bernd A Kuehlewein L Nasser F. Genetic architecture of inherited retinal degeneration in Germany: a large cohort study from a single diagnostic center over a 9-year period. Hum Mutat. (2020) 41:1514–27. doi: 10.1002/humu.24064, PMID: 32531858

[19] Wheway G Schmidts M Mans DA Szymanska K Nguyen T-MT Racher H. An siRNA-based functional genomics screen for the identification of regulators of ciliogenesis and ciliopathy genes. Nat Cell Biol. (2015) 17:1074–87. doi: 10.1038/ncb3201, PMID: 26167768 PMC4536769

[20] Zhang Q Xu M Verriotto JD Li Y Wang H Gan L. Next-generation sequencing-based molecular diagnosis of 35 Hispanic retinitis pigmentosa probands. Sci Rep. (2016) 6:32792. doi: 10.1038/srep32792, PMID: 27596865 PMC5011706

[21] Maddirevula S Alsahli S Alhabeeb L Patel N Alzahrani F Shamseldin HE. Expanding the phenome and variome of skeletal dysplasia. Genet Med. (2018) 20:1609–16. doi: 10.1038/gim.2018.50, PMID: 29620724

[22] Patel N Aldahmesh MA Alkuraya H Anazi S Alsharif H Khan AO. Expanding the clinical, allelic, and locus heterogeneity of retinal dystrophies. Genet Med. (2016) 18:554–62. doi: 10.1038/gim.2015.127, PMID: 26355662

[23] Wang Z Iida A Miyake N Nishiguchi KM Fujita K Nakazawa T. Axial Spondylometaphyseal dysplasia is caused by C21orf2 mutations. PLoS One. (2016) 11:e0150555. doi: 10.1371/journal.pone.0150555, PMID: 26974433 PMC4790905

[24] McInerney-Leo AM Wheeler L Marshall MS Anderson LK Zankl A Brown MA. Homozygous variant in C21orf2 in a case of Jeune syndrome with severe thoracic involvement: extending the phenotypic spectrum. Am J Med Genet A. (2017) 173:1698–704. doi: 10.1002/ajmg.a.38215, PMID: 28422394

[25] Khan AO Eisenberger T Nagel-Wolfrum K Wolfrum U Bolz HJ. C21orf2 is mutated in recessive early-onset retinal dystrophy with macular staphyloma and encodes a protein that localises to the photoreceptor primary cilium. Br J Ophthalmol. (2015) 99:1725–31. doi: 10.1136/bjophthalmol-2015-307277, PMID: 26294103

[26] Lai CK Gupta N Wen X Rangell L Chih B Peterson AS. Functional characterization of putative cilia genes by high-content analysis. Mol Biol Cell. (2011) 22:1104–19. doi: 10.1091/mbc.E10-07-0596, PMID: 21289087 PMC3069013

[27] Fang X Lin H Wang X Zuo Q Qin J Zhang P. The NEK1 interactor, C21ORF2, is required for efficient DNA damage repair. Acta Biochim Biophys Sin. (2015) 47:834–41. doi: 10.1093/abbs/gmv076, PMID: 26290490 PMC4581587

[28] Bai SW Herrera-Abreu MT Rohn JL Racine V Tajadura V Suryavanshi N. Identification and characterization of a set of conserved and new regulators of cytoskeletal organization, cell morphology and migration. BMC Biol. (2011) 9:54. doi: 10.1186/1741-7007-9-54, PMID: 21834987 PMC3201212

[29] Zhu Q Rui X Li Y You Y Sheng X-L Lei B. Identification of four novel variants and determination of genotype–phenotype correlations for ABCA4 variants associated with inherited retinal degenerations. Front Cell Dev Biol. (2021) 9:634843. doi: 10.3389/fcell.2021.634843, PMID: 33732702 PMC7957020

[30] Zhang L Li Y Qin L Wu Y Lei B. Autosomal recessive retinitis Pigmentosa associated with three novel REEP6 variants in Chinese population. Gene. (2021) 12:537. doi: 10.3390/genes12040537, PMID: 33917198 PMC8068040

[31] Fu L Li Y Yao S Guo Q You Y Zhu X. Autosomal recessive rod-cone dystrophy associated with compound heterozygous variants in ARL3 gene. Front Cell Dev Biol. (2021) 9:635424. doi: 10.3389/fcell.2021.635424 33748123 PMC7969994

[32] Yang L Jin X Li Y Guo Q Yang M You Y. A novel mutation located in the intermembrane space domain of AFG3L2 causes dominant optic atrophy through decreasing the stability of the encoded protein. Cell Death Dis. (2022) 8:361. doi: 10.1038/s41420-022-01160-9, PMID: 35970831 PMC9378676

[33] Kurashige T Morino H Matsuda Y Mukai T Murao T Toko M. Retinitis pigmentosa prior to familial ALS caused by a homozygous cilia and flagella-associated protein 410 mutation. J Neurol Neurosurg Psychiatry. (2020) 91:220–2. doi: 10.1136/jnnp-2019-321279, PMID: 31431468

[34] Jumper J Evans R Pritzel A Green T Figurnov M Ronneberger O. Highly accurate protein structure prediction with AlphaFold. Nature. (2021) 596:583–9. doi: 10.1038/s41586-021-03819-2, PMID: 34265844 PMC8371605

[35] Varadi M Anyango S Deshpande M Nair S Natassia C Yordanova G. AlphaFold protein structure database: massively expanding the structural coverage of protein-sequence space with high-accuracy models. Nucleic Acids Res. (2022) 50:D439–44. doi: 10.1093/nar/gkab1061 34791371 PMC8728224

[36] Dao TP Majumdar A Barrick D. Capping motifs stabilize the leucine-rich repeat protein PP32 and rigidify adjacent repeats: roles of caps in the folding of the LRR protein PP32. Protein Sci. (2014) 23:801–11. doi: 10.1002/pro.2462, PMID: 24659532 PMC4093955

[37] Pelegrini AL Moura DJ Brenner BL Ledur PF Maques GP Henriques JAP. Nek1 silencing slows down DNA repair and blocks DNA damage-induced cell cycle arrest. Mutagenesis. (2010) 25:447–54. doi: 10.1093/mutage/geq026, PMID: 20501547

[38] Chiu N Lee W Liu P-K Levi SR Wang H-H Chen N. A homozygous in-frame duplication within the LRRCT consensus sequence of CFAP410 causes cone-rod dystrophy, macular staphyloma and short stature. Ophthalmic Genet. (2022) 43:378–84. doi: 10.1080/13816810.2021.2010773, PMID: 34915818 PMC10234696

[39] Liu Q Tan G Levenkova N Li T Pugh EN Rux JJ. The proteome of the mouse photoreceptor sensory cilium complex. Mol Cell Proteomics. (2007) 6:1299–317. doi: 10.1074/mcp.M700054-MCP200, PMID: 17494944 PMC2128741

[40] Pearring JN Salinas RY Baker SA Arshavsky VY. Protein sorting, targeting and trafficking in photoreceptor cells. Prog Retin Eye Res. (2013) 36:24–51. doi: 10.1016/j.preteyeres.2013.03.002, PMID: 23562855 PMC3759535

[41] Ramamurthy V Cayouette M. Development and disease of the photoreceptor cilium. Clin Genet. (2009) 76:137–45. doi: 10.1111/j.1399-0004.2009.01240.x 19790290

[42] Bujakowska KM Liu Q Pierce EA. Photoreceptor cilia and retinal ciliopathies. Cold Spring Harb Perspect Biol. (2017) 9:a028274. doi: 10.1101/cshperspect.a028274, PMID: 28289063 PMC5629997

[43] Goetz SC Anderson KV. The primary cilium: a signalling Centre during vertebrate development. Nat Rev Genet. (2010) 11:331–44. doi: 10.1038/nrg2774, PMID: 20395968 PMC3121168

[44] Dalton S. Linking the cell cycle to cell fate decisions. Trends Cell Biol. (2015) 25:592–600. doi: 10.1016/j.tcb.2015.07.007, PMID: 26410405 PMC4584407

[45] Plotnikova OV Pugacheva EN Golemis EA. Primary cilia and the cell cycle. Methods Cell Biol. (2009) 94:137–60. doi: 10.1016/S0091-679X(08)94007-3 20362089 PMC2852269

[46] Cao J Shen Y Zhu L Xu Y Zhou Y Wu Z. miR-129-3p controls cilia assembly by regulating CP110 and actin dynamics. Nat Cell Biol. (2012) 14:697–706. doi: 10.1038/ncb2512, PMID: 22684256

[47] Watanabe Y Nakagawa T Akiyama T Nakagawa M Suzuki N Warita H. An amyotrophic lateral sclerosis-associated mutant of C21ORF2 is stabilized by NEK1-mediated hyperphosphorylation and the inability to bind FBXO3. iScience. (2020) 23:101491. doi: 10.1016/j.isci.2020.101491, PMID: 32891887 PMC7481237

