# Supplementary material for: Pathogenicity and functional analysis of CFAP410 mutations causing cone-rod dystrophy with macular staphyloma
Source: Front Med (Lausanne). 2023 Oct 12;10:1216427. doi: 10.3389/fmed.2023.1216427 (PMC10601463; doi:10.3389/fmed.2023.1216427)
Supplement: Supplementary file 1 [file Data_Sheet_1.docx]

Supplementary Material

Pathogenicity and functional analysis of *CFAP410* mutations causing cone-rod dystrophy with macular staphyloma

Shaoqing Yang ^1^, Ya Li ^2^, Lin Yang ^2^, Qingge Guo ^2^, Ya You ^2^, and Bo Lei ^1,2^*

*** Correspondence:** Bo Lei, MD, Ph.D. 7 Weiwu Road, Henan Eye Institute, Zhengzhou, Henan, 450003, China. E-mail: [bolei99@126.com](mailto:bolei99@126.com); Tel: +0371-67120925

#
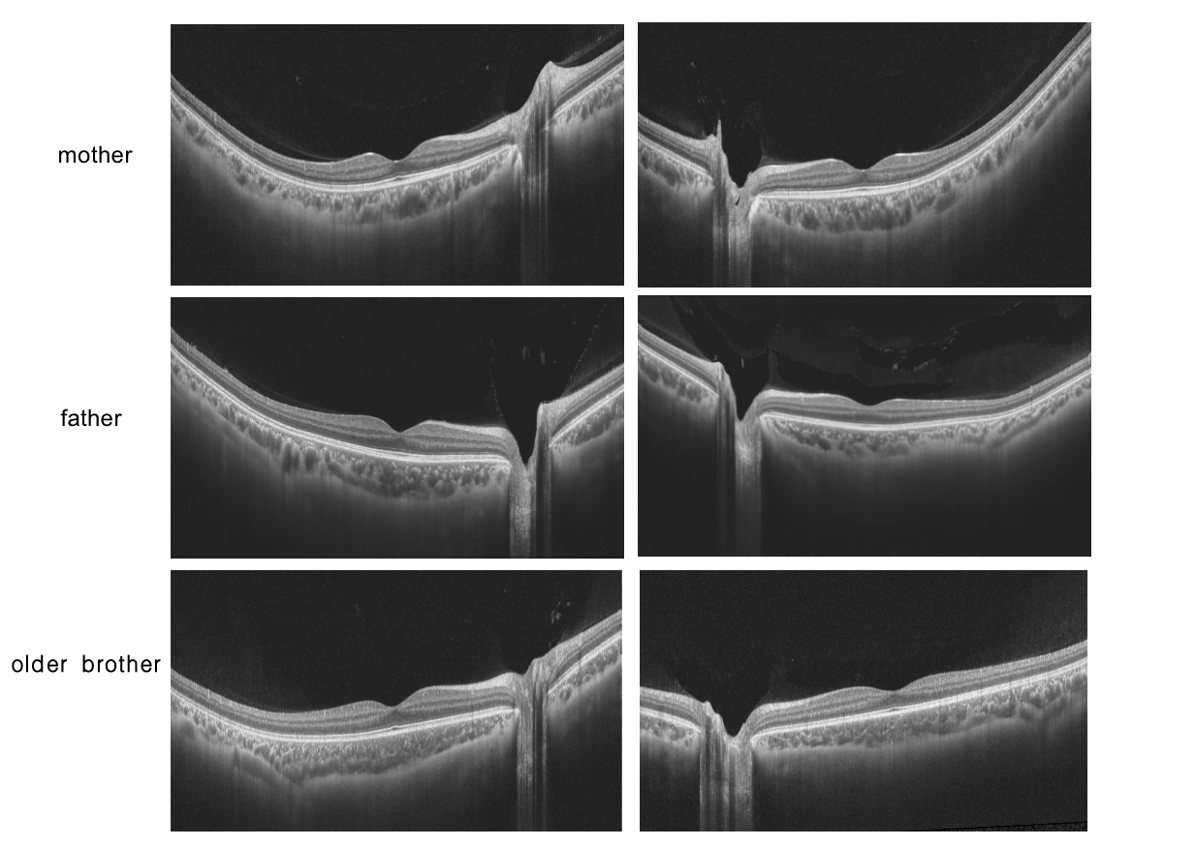
Supplementary Figures

**Supplementary Figure 1.** Ocular examinations for other members in the family. SS-OCT showed proband' parents and brother had no abnormalities in the macula.

# Supplementary Tables

| Tab.1 Other variants carried by the proband | | | | | | | |
| --- | --- | --- | --- | --- | --- | --- | --- |
| **Gene** | **Chromosomal location** | **Transcript number** | **Variable Site** | **Heterozygosity** | **Pathogenicity** | **Disease/phenotype and mode of inheritance** | |
| CEP250 | chr20-34060509 | NM_007186 | c.1062A>G (p.Glu354Glu) | Het | VUS | Cone-rod dystrophy and hearing loss type 2 (AR) |  |
| CEP290 | chr12-88505514 | NM_025114 | c.2174A>C (p.Glu725Ala) | Het | VUS | Bardet-Biedl syndrome type 14 (AR); Joubert syndrome type 5 (AR); Meckel syndrome type 4 (AR); Senior-Loken syndrome type 6(AR); Leber congenital amaurosis 10(AR) |  |
| HMCN1 | chr1-186158861 | NM_031935 | c.16759C>G (p.Leu5587Val) | Het | VUS | Age-related macular degeneration type 1 (AD) |  |
| IARS2 | chr1-220275746 | NM_018060 | c.741G>A (p.Pro247Pro) | Het | VUS | Sensorineural deafness with skeletal dysplasia (AD) |  |
| MAK | chr6-10819103 | NM_005906 | c.156+16T>C | Het | VUS | Retinitis pigmentosa type 62 (AR) |  |
| MPDZ | chr9-13119648 | NM_003829 | c.5232A>G (p.Arg1744Arg) | Het | VUS | Congenital hydrocephalus type 2 with or without ocular brain abnormalities (AR) |  |
| MYO7A | chr11-76890101 | NM_000260 | c.2293C>G (p.Leu765Val) | Het | VUS | Autosomal dominant deafness type 11(AD);Autosomal recessive deafness type 2 (AR); Usher syndrome type 1B (AR) |  |
| SLC7A14 | chr3-170201230 | NM_020949 | c.988G>A (p.Gly330Arg) | Het | VUS | Retinitis pigmentosa type 68 (AR) |  |
| USH2A | chr1-215820915 | NM_206933 | c.14740G>A (p.Glu4914Lys) | Het | VUS | Usher Syndrome type 2A (AR); Retinitis pigmentosa type 39 (-) |  |
| WFS1 | chr4-6303731 | NM_006005 | c.2209G>A (p.Glu737Lys) | Het | VUS | Cataract type 41 (AD); Non-insulin-dependent diabetes (AD); Autosomal dominant deafness type 6/14/38 (AD); Wolfram sydrome (AD) |  |

| Tab.2 In sillco pathogenicity analyses of P116L | | |
| --- | --- | --- |
| algorithm | score | prediction |
| SIFT_ | 0 | Damaging |
| Polyphen2_HDIV | 1 | Probably_damaging |
| Polyphen2_HVAR_score | 1 | Probably_damaging |
| LRT | 0 | Deleterious |
| MutationTaster | 1 | Disease_causing |
| MutationAssessor | 3.24 | Medium |
| FATHMM | 1.34 | Tolerable |
| PROVEAN | -10 | Damaging |
| VEST3 | 0.864 | Damaging |
| MetaSVM | -0.477 | Tolerable |
| MetaLR_ | 0.26 | Tolerable |
| M_CAP | 0.123 | Damaging |
| CADD | 26.5 | Damaging |
| DANN | 0.999 | Damaging |
| FATHMM_MKL | 0.834 | Damaging |
| Eigen_score | 0.62 | Damaging |
| GenoCanyon | 1 | Damaging |
| ClinPred | 0.992 | pathogenic |
| fitCons_score | 0.66 | Tolerable |
| ReVe | 0.851 | Damaging |
| GERP | 4.99 | Conserved |
| phyloP | 7.304 | Conserved |
| phastCons | 1 | Conserved |
| SiPhy_ | 15.574 | Conserved |
